# Precision medicine in cutaneous melanoma—A comprehensive review

**DOI:** 10.1111/jdv.70111

**Published:** 2025-10-13

**Authors:** Markus Reitmajer, Lukas Flatz

**Affiliations:** ^1^ Department of Dermatology University Hospital Tuebingen Tuebingen Germany; ^2^ Institute of Immunobiology, Kantonsspital St. Gallen Switzerland

**Keywords:** biomarkers, genomics, melanoma, molecular targeted therapy, Pharmacogenetics, precision medicine

## Abstract

Precision medicine aims to tailor medical treatment to the individual characteristics of each patient, beginning with detailed diagnostic assessments. In cutaneous melanoma, precision medicine has made considerable progress, with some approaches already implemented in clinical practice and guidelines. For instance, testing for BRAF mutations to guide the use of targeted BRAF/MEK inhibitor therapy is an established standard of care. Even melanoma with rarer genetic alterations, such as NTRK fusions or KIT mutations, can increasingly be treated with specific targeted therapies. These advancements have translated into significant improvements in overall survival, especially in patients with metastatic disease. Other innovative approaches, such as gene expression profiling (GEP) and the use of circulating tumour DNA (ctDNA) for monitoring treatment response, are already available. However, they are not yet part of routine clinical care or broadly recommended in guidelines. Likewise, the use of tumour‐infiltrating lymphocytes (TIL) remains limited to specialized centres, and tailor‐made mRNA vaccines are still in the investigational phase but hold great promise for the future of melanoma therapy. This review provides an overview of current and emerging approaches in precision therapy and diagnostics for melanoma, encompassing key areas such as molecular and genomic profiling, advanced imaging techniques, biomarker‐based stratification and AI‐driven technologies.

## Graphical abstract

Precision medicine tailors treatment to each patient, beginning with detailed diagnostics. In melanoma, it has advanced considerably: Some methods are in practice and guidelines, while others are commercially available but not routine. This review highlights current and emerging approaches, including molecular profiling, imaging, biomarkers and AI.

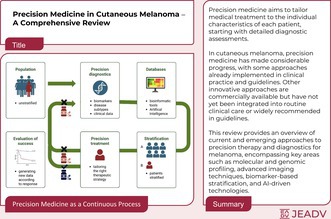

**Why was the study undertaken?**

The objective of this study was to conduct a comprehensive literature review on the current status and clinical application of precision medicine in the treatment of melanoma.

**What does this study add?**

This study systematically highlights recent advances in precision medicine for melanoma, including the clinical implementation of molecular profiling, artificial intelligence (AI), gene expression profiling (GEP), as well as promising future treatments.

**What are the implications of this study for disease understanding and/or clinical care?**

Precision medicine is no longer just a vision of the future. It is an emerging and valuable concept that is already showing remarkable results in the treatment of melanoma. In this review, we aim to introduce the concept and provide an overview of the current state of research and clinical application.

## INTRODUCTION

**FIGURE 1:**
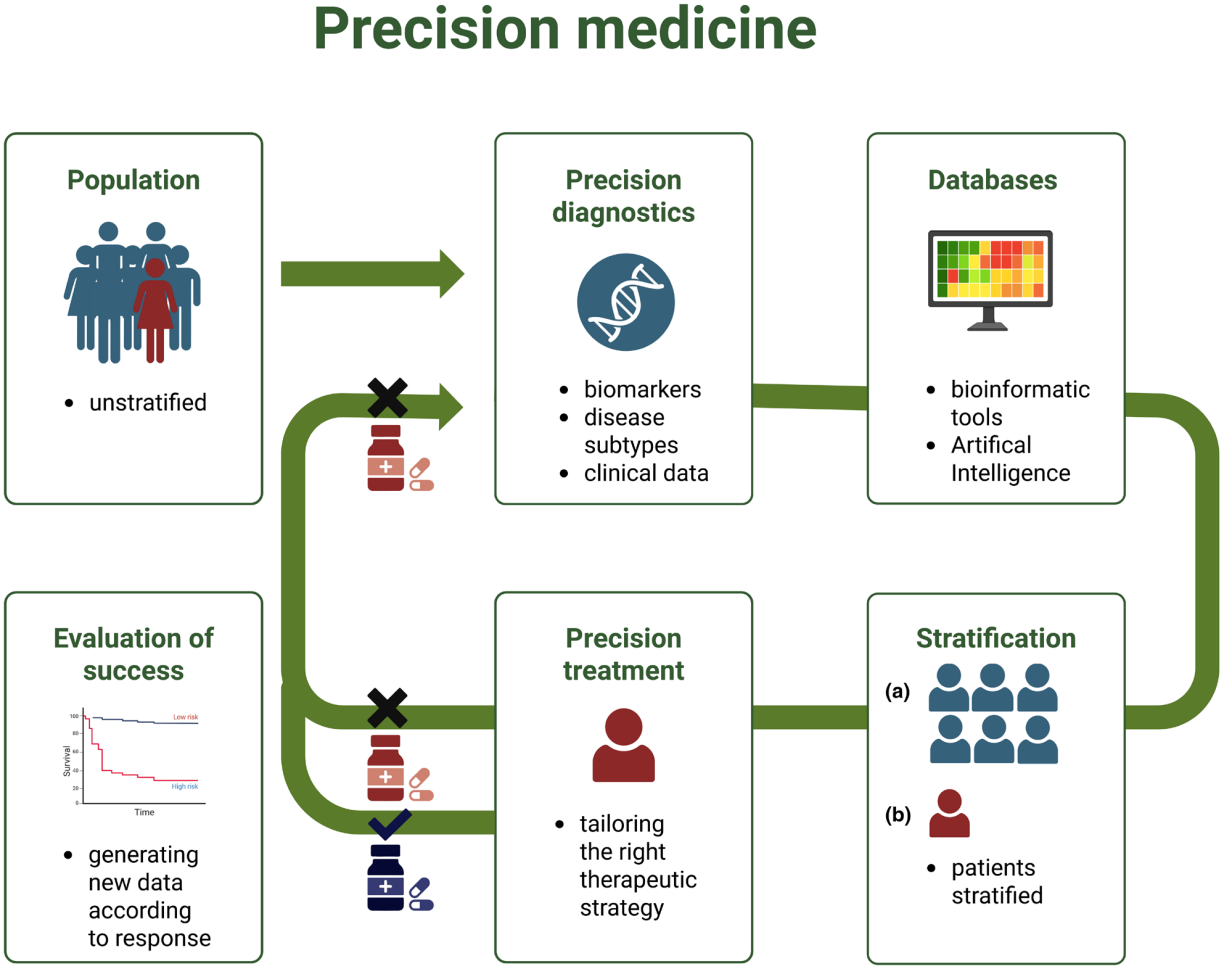
Precision medicine. This figure illustrates precision medicine as a continuous process, rather than a one‐way pipeline. The process does not end at treatment initiation: In the case of treatment, reassessment through updated diagnostics and renewed data matching is essential to guide further therapy decisions. The figure was created using the web‐based figure design platform BioRender (BioRender Inc., Toronto, Canada).

Precision medicine, also known as personalized medicine, is a widely discussed concept not only in medical literature but increasingly also in the political and societal spheres.^1^ The idea behind it is to move away from a ‘one‐size‐fits‐all’ approach in healthcare and instead tailor prevention, diagnosis and treatment strategies to the individual characteristics of each patient.^2^ The concept is characterized by its dynamic nature and should be understood as a continuous process, rather than a one‐way pipeline. Following initial diagnostics and the integration of patient‐specific data with existing databases, patients are stratified, and an individualized therapeutic strategy is selected. However, this process should not conclude with the initiation of therapy. In cases of treatment failure or suboptimal response, reassessment through updated diagnostics and renewed integration of molecular and clinical data is essential (Figure 1).

In 2015, the Health Ministers of the European Union defined in their council conclusion the concept of personalized medicine as ‘a medical model using characterization of individuals' phenotypes and genotypes (e.g. molecular profile, medical imaging, lifestyle data) for tailoring the right therapeutic strategy for the right person at the right time’.^1^ The overlap between the terms ‘precision medicine’ and ‘personalized medicine’ is fluid and not clearly defined. According to the National Research Council, the term ‘precision medicine’ is preferable to the older term ‘personalized medicine’, as this may be misinterpreted to imply that entirely individualized therapies are developed for each patient. In contrast, ‘precision medicine’ more accurately reflects current clinical practice, which involves the use of molecular diagnostics to stratify patients and tailor therapies accordingly.^3^ However, the concept of shared medical decision‐making, which is an important aspect of palliative oncologic care, engages patients and healthcare professionals in choosing options that reflect patient preferences in a highly personalized manner.^4^, ^5^ Both terms continue to be used in clinical practice as well as in scientific and policy discourse, including in the context of this article.

In the following, we review the extent to which precision medicine has already been included in the diagnosis and treatment of cutaneous melanoma, and discuss potential future applications in clinical practice and guideline development. To provide orientation in this broad field, we used the TNM structure of the eighth edition of the American Joint Committee on Cancer (AJCC) classification to categorize the various approaches in precision diagnostics, concluding with an overview of precision therapies.

## PRIMARY TUMOUR THICKNESS (T) AS A POTENTIAL TARGET FOR PRECISION MEDICINE AND THE ROLE OF ARTIFICIAL INTELLIGENCE (AI) IN ACHIEVING IT

Cutaneous melanoma is among the most prevalent cancers in fair‐skinned populations, particularly in regions such as Europe, Australia and the United States. In 2020, approximately 325,000 new cases and 57,000 deaths worldwide were attributed to cutaneous melanoma.^6^, ^7^ If current trends continue, the global burden of melanoma is projected to rise to 510,000 new cases (an approximate 50% increase) and 96,000 deaths (an approximate 68% increase) by 2040, posing a significant challenge to healthcare systems worldwide.^8^ However, the treatment costs for advanced stage IV melanoma far exceed those of stage I melanoma. In a 2023 study from Ireland, the cost of managing melanoma diagnosed at stage IV was more than 25 times higher than that of a case diagnosed at stage IA, highlighting the importance of early detection from a public health perspective.^9^

Early detection through population‐based skin cancer screening programmes has been discussed in several countries with the aim of identifying primary melanomas with minimal tumour thickness.^10^, ^11^, ^12^ However, the evidence on the effectiveness of mass screening for melanoma, such as it is currently conducted according to guidelines in Germany, is limited.^11^, ^13^ Instead, most countries including the United States of America, the United Kingdom and Australia recommend a more targeted approach, which involves regular skin cancer screening programmes focused on high‐risk groups or on patients at increased risk, which can also be seen as precision medicine, following the European Union's definition of ‘the right person at the right time’.^1^, ^12^ The idea behind all these approaches is to reduce mortality and the financial burden associated with the high‐cost systemic treatment of advanced melanoma stages by detecting the disease at an early stage, when the tumour has minimal vertical thickness.^12^, ^14^

The eighth edition of the AJCC classification defines the stages and therefore prognosis of melanoma.^15^ The vertical primary tumour thickness and ulceration (T), as part of the AJCC classification for determining the stage of melanoma, is a relevant prognostic marker and is commonly measured by Breslow depth.^15^, ^16^ Thicker tumours are associated with a higher risk of metastasis and poorer outcomes, underscoring the importance of early detection when the primary tumour is still thin.^17^, ^18^ Artificial intelligence (AI) is increasingly being explored for its potential in the early detection of melanoma and for individually personalized longitudinal monitoring of moles, thereby enhancing the accuracy and efficiency of skin cancer screening.^19^, ^20^, ^21^, ^22^ AI systems have the potential to flag malignant lesions and save these flagged lesions for comparison during future skin screenings. This type of surveillance has the ability to detect subtle changes that might be overlooked by clinicians.^22^, ^23^ The potential superiority of AI algorithms over experienced, board‐certified dermatologists and the associated question of whether dermatologist‐led dermoscopy remains essential, is unresolved.^24^ However, current evidence suggests that AI should be viewed as an adjunctive tool that supports clinical decision‐making rather than as a standalone gold standard.^25^, ^26^ Limitations of AI systems have been demonstrated, particularly in scenarios insufficiently represented in the used training datasets, such as image artefacts or ulcerations.^27^ AI is not only being applied to the early detection of primary tumours with minimal thickness but also to the non‐invasive assessment of tumour thickness. Raster Scanning Optoacoustic Mesoscopy (RSOM) is a method for non‐invasively determining tumour thickness at single‐cell resolution and may gain increasing relevance with the growing adoption of neoadjuvant immune checkpoint inhibitor (ICI) therapy.^28^

## LYMPH NODE STATUS (N) TODAY AND ITS FUTURE ROLE

Lymph node status (N) is a key prognostic factor in clinical practice, indicating whether malignant cells have already spread from the primary melanoma to nearby lymph nodes, assessed through a sentinel lymph node biopsy (SLNB).^15^, ^29^ Since the early 1990s, lymph node status has served as a pivotal parameter in clinical decision‐making, particularly for determining the necessity of completion lymph node dissection and indication for adjuvant systemic therapy.^30^, ^31^ According to the current guidelines of the European Association of Dermato‐Oncology (EADO) and the American Society of Clinical Oncology (ASCO), a sentinel lymph node biopsy (SLNB) is consistently offered for a tumour thickness of ≥1.0 mm.^32^, ^33^ In patients with a tumour thickness of 0.8–1.0 mm, a range of additional factors, such as ulceration, patient age and mitotic rate, must be considered in the decision‐making process.^32^, ^33^, ^34^, ^35^ However, patients are exposed to perioperative risks from a procedure that confirms a negative sentinel lymph node in approximately 80%–85% of cases. Additionally, the anatomical location of the lymph node, for example, lymph nodes located in the head and neck region, can make the surgical procedure more difficult and associated with higher risk.^36^, ^37^ New methods such as gene expression profiling (GEP) and immunohistochemistry (IHC), which provide personalized risk scores based on gene or protein expression from the primary tumour tissue, are currently being considered for potential implementation. These tests can stratify patients into low‐ and high‐risk groups. In the case of classification into a high‐risk group, further imaging diagnostics and initiation of adjuvant therapy can proceed without prior sentinel lymph node biopsy (SLNB). Conversely, patients classified as low‐risk could be managed with a surveillance‐only approach.^38^, ^39^ With MelaGenix (Neracare), the Merlin Assay (SkylineDx) and DecisionDx‐Melanoma (Castle Biosciences), three tests based on GEP are commercially available but not yet implemented into clinical guidelines.^32^, ^33^, ^38^, ^40^, ^41^, ^42^ The same applies to the commercially available IHC‐based stratification test ImmunoPrism (Neracare).^32^, ^33^, ^43^ Furthermore, the use of AI to develop deep learning models based on existing clinical data and conventional haematoxylin and eosin staining of the primary tumour for predicting the potential risk of lymph node metastasis offers a promising tool for clinical decision‐making.^44^

## PRECISION MEDICINE APPROACHES IN IMAGING AND BIOMARKER‐BASED MONITORING OF DISTANT METASTASES (M)

Regarding the final component of the eighth AJCC TNM classification, the presence of distant metastases (M), various new modalities have also been considered for melanoma. An important example is ^18^F‐fluorodeoxyglucose positron emission tomography–computed tomography (PET‐CT), which is already used in clinical practice.^32^, ^45^ PET‐CT significantly improves the ability to distinguish whether lesions seen on conventional computed tomography (CT) scans represent active metastases or are merely residual tissue.^46^ This enables personalized decisions regarding the continuation or discontinuation of systemic therapy as well as potentially planned metastasectomy.^47^, ^48^

Circulating tumour DNA (ctDNA), measured in plasma, has also already been investigated in various studies as a promising biomarker.^49^, ^50^, ^51^, ^52^ ctDNA can be detected in most tumour patients, particularly in those with advanced disease. Technological advances in next‐generation sequencing (NGS) approaches have significantly improved the sensitivity for detecting even low levels of ctDNA in plasma.^53^, ^54^ Monitoring ctDNA in patients with advanced melanoma has proven to be complementary, and in some cases superior, to other biomarkers (S100 and LDH), and it correlates well with metabolic tumour burden compared in PET‐CT scans.^52^ ctDNA assays can be conducted using minimally invasive blood sampling and thus offer a practical approach for integration into clinical practice.^55^ The underlying idea is to enable a non‐invasive method for both therapy monitoring and surveillance, capable of detecting disease progression, treatment response or recurrence.^55^, ^56^ Moreover, ctDNA analysis can provide insights into tumour‐specific characteristics such as somatic mutations and methylation profiles.^57^, ^58^ Furthermore, the information obtained through ctDNA analysis, including somatic mutations and methylation patterns, may support the development of new treatment strategies in the context of therapy resistance. A comprehensive overview of all diagnostic approaches presented is provided in Table 1.

**TABLE 1:** Overview of diagnostic modalities and their integration into precision oncology approaches for melanoma.

Eighth AJCC TNM‐classification	Precision medicine in melanoma diagnostic	Commercially available	Implemented in clinical practice	Impact on therapy and/or availability of tailored treatments
T	(Risk‐adapted) skin cancer screening (dermoscopy)	Yes	Yes	Yes
	AI‐assisted image analysis (decision support)	Yes	Partial	Partial
	AI standalone diagnostic tool	No	No	No
N	SLNB	Yes	Yes	Yes
	GEP	Yes	No	Yes
	ctDNA	Yes	No	No
M				
	PET–CT	Yes	Yes	Yes
	Mutation analysis (NGS)	Yes	Yes	Yes
	HLA genotyping	Yes	Yes	Yes

*Note*: Green, Yes; Yellow, Partial; Orange, No.The table summarizes diagnostic approaches in precision medicine for melanoma across the TNM components.

Abbreviations: AI, artificial intelligence; AJCC, American Joint Committee on Cancer; ctDNA, circulating tumour DNA; GEP, gene expression profile; M, metastases; N, lymph nodes; NGS, Next‐Generation Sequencing; PET‐CT, Positron Emission Tomography–Computed Tomography; SLNB, sentinel lymph‐node biopsy; T, primary tumour.

## PRECISION MEDICINE IN THE TREATMENT OF CUTANEOUS MELANOMA

For thin melanoma, surgical excision is the treatment of choice, followed by regular follow‐up.^32^, ^33^ Historically, advanced cutaneous melanoma was associated with a poor prognosis and limited treatment options, primarily relying on a one‐size‐fits‐all approach using chemotherapy agents such as dacarbazine or carboplatin/paclitaxel.^59^, ^60^ Prior to 2010, survival rates for patients with stage IV melanoma were poor, with a 5‐year survival reported at approximately 5%.^61^ In the 10‐year follow‐up of the KEYNOTE‐006 study and the CheckMate 067, patients treated with first‐line combined immune checkpoint inhibition (ICI) exceeded 50%.^62^, ^63^ In real‐world cohorts, the 5‐year survival rates are reported to range between 32% and 36%.^64^, ^65^

However, the remarkable improvement in overall survival in stage IV melanoma is not solely attributable to the implementation of immune checkpoint inhibitors in standard treatment (overview in Table 2). Compared to many other tumour types, melanoma exhibits a high tumour mutational burden, with certain mutations such as those in BRAF, NRAS and KIT occurring at relatively high frequencies.^66^ BRAF and NRAS play a pivotal role as messenger molecules in the mitogen‐activated protein kinase (MAPK) pathway, which transmits growth signals from the cell surface to the nucleus.^67^ Mutations in this signalling pathway can lead to increased, uncontrolled cell proliferation, as seen in melanoma.^67^, ^68^ Approximately half of all melanomas have such a driver mutation in the BRAF gene, and around 15%–20% show a mutation in the NRAS gene.^69^, ^70^ In the context of precision medicine, mutation status is already routinely assessed, evaluated and used as a therapeutic target in clinical practice.^32^, ^33^ While three potent and commercially available BRAF/MEK inhibitor combinations (Dabrafenib with Trametinib, Encorafenib with Binimetinib, and Vemurafenib with Cobimetinib) are currently available, the treatment of NRAS‐mutated/BRAF‐wildtype melanoma remains more challenging.^71^, ^72^, ^73^ In contrast to BRAF inhibition, NRAS is considered an ‘undruggable’ target, as it lacks an easily accessible binding pocket for small molecules, making the development of specific inhibitors challenging.^74^ To provide options for BRAF negative patients, pan‐RAF kinase inhibitors, including Belvarafenib, Exarafenib and Naporafenib, are currently under evaluation. These agents target the entire RAF kinase family and have demonstrated preliminary antitumor efficacy in phase 1 studies.^75^, ^76^, ^77^

**TABLE 2:** Overview of therapy options in melanoma according to molecular/genetic alterations.

Mutation	Frequency	Typical subtype association	Targeted therapy	Implemented in clinical practice
BRAF V600E/K	~40%–50%	Cutaneous melanoma younger patients	Dabrafenib/Trametinib, Encorafenib/Binimetinib and Vemurafenib/Cobimetinib	Commercially available
NRAS	~15%–20%	Cutaneous melanoma often aggressive	Pan‐RAF kinase inhibitors (phase II studies)	Not commercially available
c‐KIT	~1%–4%	Mucosal and acral melanoma	KIT‐inhibitor (Imatinib)	Commercially available
NTRK Fusions	<1%	Spitzoid, paediatric melanoma	TRK (Larotrectinib)	Commercially available
High TMB	~30%–50% in cutaneous subtype; lower in others	UV‐induced melanoma	Association with high ICI response rates	Potential biomarker, but not used for decision‐making
PD‐L1 Expression	Variable (~20%–50%)	All melanoma subtypes, heterogeneous expression		
HLA‐A02:01	~40%–50% (in Caucasian populations)	/	Tebentafusp	Not approved for cutaneous melanoma; currently in Phase III trial

*Note*: Green, Commercially available; Orange, Not commercially available or not used in clinical practice. The table summarizes therapy options in melanoma regarding their molecular/genetic alterations.

Abbreviations: ICI, immune‐checkpoint‐inhibitors; PD‐L1 expression, programmed death‐ligand 1 expression; TMB, tumour mutational burden; UV, ultraviolet radiation exposure.

Apart from the MAPK pathway, additional potential therapeutic targets have been identified, although they occur at significantly lower frequencies. Approximately 1%–4% of all melanomas harbour a c‐KIT mutation, with increased prevalence in patients with acral and mucosal melanomas.^78^, ^79^ Imatinib, a tyrosine kinase inhibitor, has demonstrated high efficacy, with overall disease control rates of 77% in patients with c‐KIT mutations.^80^ Therefore, melanoma subtypes such as acral and mucosal, which typically exhibit lower response rates to other therapies, should be sequenced for potential c‐KIT aberrations. Furthermore, fusions in the neurotrophic tyrosine receptor kinase (NTRK) genes, known for their oncogenic potential in various solid tumours, particularly in fibrosarcoma, breast cancer and also melanoma, have been identified as potential therapeutic targets.^81^, ^82^ Larotrectinib, as tropomyosin receptor kinase inhibitor, is the first representative of a new class of drugs specifically approved for the treatment of cancers with NTRK gene fusions and is not limited to a specific type of cancer.^82^, ^83^ This novel class of drugs exemplifies the concept of precision medicine, as it moves beyond conventional definitions and specifically targets molecular biomarkers.

In contrast to targeted therapy of the MAPK pathway, c‐KIT mutations and fusions in NTRK, ICI are considered to have no specific requirements and can be used in the presence of either a BRAF‐ or an NRAS mutation. While there are prognostic markers associated with a higher response rate, for instance, the tumour mutational burden, the Programmed Death‐Ligand 1 status (PD‐L1) or specific tumour neoantigens, these have not yet been established in clinical practice as criteria for initiating or not initiating immunotherapy.^84^, ^85^, ^86^ Instead, the timing of ICI application has become a key focus in enhancing therapeutic efficacy, underscoring the value of ‘the right time’ within the paradigm of personalized medicine. The SWOG study (neoadjuvant/adjuvant pembrolizumab in resectable stage IIIB to IV melanoma) and the NADINA study (neoadjuvant ipilimumab/nivolumab in resectable, macroscopic stage III melanoma) showed both benefits in event‐free survival when initiating ICI before surgery.^87^, ^88^ The underlying idea is that resection of the bulk of the tumour also removes tumour‐infiltrating lymphocytes (TIL), which may represent potential antitumor T cells that would otherwise proliferate in response to ICI treatment. These TIL can also be directly obtained from patient‐derived tumour material through surgical resection, followed by ex vivo stimulation via co‐culture with cytokines and expansion to generate a personalized TIL infusion product.^89^ While the U.S. Food and Drug Administration (FDA) has approved Lifileucel as the first TIL therapy for this purpose in the USA, it has not yet been approved by the European Medicines Agency (EMA) in Europe.^90^ Lifileucel demonstrated a high overall response rate (49%) among patients with advanced melanoma after failure of first‐line therapy and could therefore be considered as a potential second option in future melanoma therapy to further improve the survival of these patients.^89^, ^90^

In the context of personalized or precision medicine, mRNA vaccines could represent a future key innovation that deserves particular attention. The potential of mRNA vaccines was demonstrated during the COVID‐19 pandemic and has since been increasingly explored for applications in cancer therapy.^91^ The concept is to deliver synthetic mRNA, which encodes for tumour‐specific antigens into the patient's cells, typically via lipid nanoparticles, where it is translated into antigenic proteins. These proteins are then presented on the cell surface via MHC molecules, thereby activating both CD8^+^ cytotoxic T cells and CD4^+^ helper T cells for a targeted immune response against cancer cells expressing the same antigens.^92^, ^93^ Currently, two different approaches are under investigation. In one approach, tumour‐specific antigens are used to produce mRNA vaccines; for example, in melanoma, antigens such as Melan‐A (Melanoma Antigen), MAGE‐A3 (Melanoma Associated Antigen 3) and NY‐ESO‐1 (New York Esophageal Squamous Cell Carcinoma 1) are utilized. In the other approach, patient‐derived tumour tissue is collected and subsequently analyzed for potentially immunogenic tumour antigens. The goal of this more personalized strategy is to create individual tumour antigen profiles, which serve as the basis for developing tailored mRNA vaccines.^94^ No mRNA vaccines for cutaneous melanoma have been approved by the FDA or EMA yet. However, several clinical trials are ongoing, and the first long‐term results from the KEYNOTE‐942 study have been published. In this randomized, phase 2b adjuvant trial, the combination of mRNA‐4157 (a personalized mRNA vaccine) and pembrolizumab was compared to pembrolizumab monotherapy in patients with completely resected stage IIIB–IV melanoma. After 2.5 years, recurrence‐free survival showed a significant 49% risk reduction in the combination group, and a trend for improved overall survival was observed.^95^, ^96^ While no mRNA vaccines for melanoma are currently commercially available, approvals are expected within the next years, potentially transforming the treatment landscape for melanoma.

The current state of development for the so‐called T cell engagers (BiTEs, bispecific T cell engagers) is more advanced. For uveal melanoma, a candidate (Tebentafusp) has already been approved for patients with the HLA type HLA‐A02:01. BiTEs are artificially engineered antibodies with two binding sites: one for a tumour antigen on the cancer cell and one for the CD3 receptor on the T cell, aiming to bring T cells into close proximity with tumour cells and induce their killing.^97^ In the case of Tebentafusp, the target is the tumour antigen gp100 presented on HLA‐A02:01, which makes HLA typing a necessary step before initiating therapy. At a minimum follow‐up of 36 months, Tebentafusp demonstrated an overall survival benefit in patients with metastatic uveal melanoma compared to the investigator's choice of single‐agent pembrolizumab, ipilimumab or dacarbazine.^98^ Given gp100 as the target, the use of Tebentafusp is now being evaluated for cutaneous melanoma in the TEBE‐AM trial (NCT05549297), a Phase 3 study of Tebentafusp with or without pembrolizumab in advanced cutaneous melanoma.

## CONCLUSIONS

The field of precision medicine in cutaneous melanoma diagnosis and treatment has undergone substantial advancements in recent years, with some concepts already translated into clinical practice and others currently under investigation. The increasingly precise understanding of molecular pathophysiology, such as mutation analysis of BRAF/NRAS status, holds significant therapeutic importance. Also, rare mutations such as c‐KIT or NTRK gene fusions are already clinically relevant in selected cases. The results of these efforts are reflected in the significant improvement in overall survival for patients with advanced melanoma. Other technologies, such as the use of AI for skin cancer detection or GEP assays for patient risk stratification, are currently being implemented, while other approaches like mRNA vaccines are still in the stage of clinical trials. However, precision medicine extends beyond developing new drugs and improving technologies. It also involves the optimal use of existing resources, tailored to the right patient at the right time. In this context, approaches like the neoadjuvant usage of ICI therapy for cutaneous melanoma have already been successfully incorporated into clinical practice.

On initial consideration, evidence‐based guidelines and precision medicine appear to have fundamentally different orientations. While clinical guidelines aim to provide standardized directives, including instructions and recommendations to ensure consistency in treatment and diagnostic procedures, precision medicine may take a contrasting path.^2^, ^99^ Rather than applying standards, it adopts an individualized strategy, tailoring diagnostics, therapeutic interventions and preventive measures to the unique genetic, molecular, environmental and lifestyle characteristics of patients as a ‘tailor‐made approach’.^2^, ^100^, ^101^ However, these two clinical approaches are not strictly opposed to each other. In fact, the concepts of precision medicine are increasingly integrated into clinical guidelines.^33^, ^102^, ^103^, ^104^ Nevertheless, this requires that databases are accessible across institutions, which remain a major challenge due to commercial and proprietary barriers. Overcoming these barriers is essential to unlock the full potential of precision medicine.

## AUTHOR CONTRIBUTIONS

L.F. and M.R.: writing – original draft preparation, conceptualization, methodology, visualization, writing – review and editing. All authors approved the final version of the article.

## FUNDING INFORMATION

None.

## CONFLICT OF INTEREST STATEMENT

MR received funding as part of the Clinician Scientist Program of the University of Tuebingen (application no. 523‐0‐0) and travel support from Almirall Hermal, Galderma and Pierre‐Fabre, all outside the submitted work. LF received grants from Hookipa Pharma, Swiss Cancer League, German Research Foundation, Immunophotonics, Mundipharma. LF received consulting fees from Philogen and support for attending meetings or travel from Philogen, Hookipa Pharma. LF participates on the board for the University of Basel (TIL trial, unpaid) and is the founder of Hookipa Pharma, Schmelzberg, Humion and Abtherix—all outside the submitted work.

## ETHICAL APPROVAL

Not applicable.

## ETHICS STATEMENT

Not applicable.

## Data Availability

Data sharing is not applicable to this article as no new data were created or analysed in this study.
